# Effect of Chewing Gum on Gastrointestinal Function Recovery After Surgery of Gynecological Cancer Patients at Rajavithi Hospital: A Randomized Controlled Trial

**DOI:** 10.31557/APJCP.2020.21.3.761

**Published:** 2020-03

**Authors:** Arphamart Nanthiphatthanachai, Putsarat Insin

**Affiliations:** 1 *Department of Obstetrics and Gynecology, Rajavithi Hospital, *; 2 *College of Medicine, Rangsit University, Bangkok, Thailand. *

**Keywords:** Gum-chewing, gynecological cancer surgery, gastrointestinal function recovery, postoperative bowel ileus

## Abstract

**Objective:**

To evaluate the efficacy of postoperative gum-chewing compare with routine postoperative care on the recovery of gastrointestinal function after comprehensive surgical staging for gynecological cancer.

**Materials and Methods:**

A total of 82 patients who underwent comprehensive surgical staging for gynecological cancer at Rajavithi Hospital between October 1st, 2018 and June 30th, 2019 were randomly allocated into two groups: Gum-chewing group (n=40) and control group (n=42). In the gum-chewing group, patients were assigned to chew sugar-free gum for 30 minutes starting from the first postoperative morning then every 8 hours until the first passage of flatus. In the control group, patients have received routine postoperative care. The primary endpoint was time to first flatus after surgery. The secondary endpoints were time to first bowel sound, time to first defecation, time to first walk, postoperative analgesia and anti-emetic drug requirement, ileus symptoms, length of a hospital stay, and potential adverse events of gum-chewing, including dry mount, choking, and aspiration.

**Results:**

Chewing gum was statistically significant in reducing time to first flatus compared with routine postoperative care (median 24.7 (range 2.2-86.5) vs 35.4 (range 7.2-80.9) hours, p=0.025). The length of a hospital stay was also significantly shorter in the gum-chewing group (median 3.0 (range 1.0-8.8) vs 3.5 (range 1.8-50.0) days, p=0.023). There were no significant differences in time to first bowel sound, time to first defecation, time to first walk, postoperative analgesia and anti-emetic drug requirement, and ileus symptoms between both two groups. No adverse events related to postoperative gum-chewing were observed.

**Conclusion:**

Gum-chewing was associated with early recovery of gastrointestinal function in patients undergoing surgery for gynecological cancer. It is an inexpensive and physiologic intervention that appears to be reasonably safe and should be recommended as an adjunct in postoperative care of gynecological cancer surgery.

## Introduction

Cancer is a major public health concern and is the second leading cause of death worldwide, after cardiovascular disease (Bray et al., 2018). Although breast cancer is the most common cancer occurring in female, gynecological cancers have also affected them. Gynecological cancers are cancers that originate in the female reproductive tracts. The five main types of gynecological cancers are cervical, ovarian, uterine, vaginal, and vulvar. The incidence rate of gynecological cancers among women varied by cancer type and ethnicity. In 2019, gynecological cancers accounted for approximately 12.2% (109,000 out of 891,480) of all new cancers diagnosed among women in the United States and contribute to 33,100 annual deaths (Siegel et al., 2019). Among women in Thailand, an estimated overall incidence of gynecological cancer is approximately 10% with the most common cancer is cervical cancer (14.4 cases per 100,000), followed by ovarian cancer (6.0 cases per 100,000), and uterine cancer (4.3 cases per 100,000) (Wilailak and Lertchaipattanakul, 2016). Although the overall incidence rate is declining, the number of cancer survivors continues to grow because of improvements in the treatment of these diseases. However, these patients have experienced treatment side effects and decrease quality of life. Therefore, the physical and psychological sequelae of cancer treatment are well recognized and becoming an interesting issue as a role in the improvement of gynecological cancer care (Campbell et al., 2019). 

Surgery remains the mainstay of gynecological cancer treatment, either primarily surgery for tumor staging and debulking, or secondarily for treatment of complications related to prior therapy. The surgical procedures may range from staging, debulking, total/radical hysterectomy, unilateral/bilateral salpingo-oophorectomy, omentectomy, and lymph node removal. These procedures were performed through a large abdominal incision and were associated with prolonged hospitalization and significant morbidity. Post-operative bowel ileus (POBI) which is a delay in the return of normal bowel function presenting in the passage of flatus and feces is one of the most important factors affecting early recovery and hospital discharge in these patients (Kehlet and Holte, 2001). The incidence of POBI ranged from 10.6 to 50.0% in patients treating with comprehensive surgical staging for gynecological cancers (Fujita et al., 2005; Tabata et al., 2010). Previous studies have shown that a post-operative bowel recovery time of 12 to 24 hours is required for the small bowel, 24 to 48 hours for the stomach, and 3 to 5 days for the large bowel (Mattei and Rombeau, 2006). Delayed gastrointestinal function recovery may cause abdominal distention, abdominal pain, nausea, vomiting, and even intestinal obstruction in severe cases. Consequently, these made a prolonged hospital stay and associated with an increased risk of hospital-acquired infections, deep vein thrombosis, pulmonary disease, and total hospital costs (Johnson and Walsh, 2009; Asgeirsson et al., 2010). For these reasons, a variety of procedures have been investigated and implemented for the management of bowel function recovery.

Currently, enhanced recovery after surgery (ERAS) protocol which is a collection of best practices implemented with a goal standardizing perioperative care is now developed (Ljungqvist et al., 2017). ERAS was initially created in Europe and then largely adopted by colorectal surgeons in the United States. Due to significant improvements in patient care, it has continued to develop across various surgical fields, including urology and surgical oncology. For gynecologic oncology, ERAS guidelines were published in 2016 and have been shown to have multiple benefits for post-operative gynecological cancer patients that including decreased surgical complications, shorter length of hospital stays, and lower cost (Nelson et al., 2016b; Nelson et al., 2016a). One issue of the ERAS measures is considered effective in stimulating bowel movement. Several different interventions have been used to manage bowel function, including adequate pain control, prokinetic drug, coffee consumption, gum-chewing, and supportive strategies including nasogastric decompression, early ambulation, and early oral feeding (Charoenkwan et al., 2007; Purkayastha et al., 2008; Traut et al., 2008; Johnson and Walsh, 2009; Gungorduk et al., 2017; Nelson et al., 2019). However, these guidelines were included many different recommendations from weak to strong based on the level of evidence. Thereby, it is still controversy and no strong recommendation for gynecological cancer surgery.

Gum-chewing mimics food intake and is considered a kind of sham feeding. With the theory of Pavlov that sham feeding had a similar effect on the gastrointestinal tract as normal feeding (Konturek, 2003). The thought, sight, smell, taste, and chewing of food induce the vagal nerve to release gastrointestinal hormones. Such the physiologic mechanism for the enhanced recovery of bowel function by gum-chewing is assumed to be the activation of the cephalic-vagal pathway, which is stimulating intestinal myoelectric activity in an attempt to counteract the activation of the gastrointestinal u opioid receptor. It also seems to be an indirect effect by triggering the release of gastrointestinal hormones and increasing the secretion of saliva and pancreatic juice (Arosio et al., 2004). Therefore, gum-chewing has been suggested as an attractive alternative early post-operative feeding for the prevention of POBI. 

To date, several studies have been reported regarding the effect of gum-chewing in gastrointestinal function recovery for gynecological surgery. For instance, a study in gynecological cancer surgery reported that perioperative use of gum-chewing had a positive effect on the incidence of POBI (36% vs 15%) and length of hospital stay (one-day reduction) in patients undergoing surgical staging (Ertas et al., 2013). Likewise, a randomized trial showed the rate of nausea and POBI were decreased by 37.2% and 100% with the administration of gum-chewing after benign gynecological surgery (Jernigan et al., 2014). Also, a similar result has been observed after cesarean delivery that gum-chewing has shortened mean time intervals to normal bowel sound (10.9 vs 15.6 hours), the passage of flatus (17.9 vs 24.4 hours), and discharge from the hospital (40.8 vs 50.0 hours) (Abd-El-Maeboud et al., 2009). 

Furthermore, recently meta-analyses also showed that gum-chewing could significantly improve bowel function in patients after colorectal resection, cesarean section, and gynecological surgery (Purkayastha et al., 2008; Craciunas et al., 2014; Xu et al., 2018). However, these investigations still have some limitations, such as high heterogeneity (e.g. difference characteristic of patients, type of surgery, type of gum, definition and measurement scale of post-operative ileus), and a relatively small number of sample size. Additionally, there is insufficient data to determine the effect of gum-chewing on the recovery of gastrointestinal function after gynecological cancer surgery. Therefore, this study was undertaken to address two issues concerning gum-chewing in gynecological cancer patients who undergo comprehensive surgical staging surgery whether it is safe and effective in gastrointestinal function recovery for these patients. 

The aim of the present study was to test the hypothesis that the administration of gum-chewing after gynecological cancer surgery was safe and would hasten gastrointestinal function recovery in terms of time to first flatus, time to first bowel sound, time to first defecation, and time to first walk, including decreasing the length of hospital stay.

## Materials and Methods

*Study design and settings*

The present study was a randomized controlled trial that included 86 patients undergoing elective comprehensive surgical staging surgery for gynecological cancers during the period from 1st October 2018 to 30th June 2019 in the Department of Obstetrics and Gynecology, Rajavithi Hospital, Bangkok, Thailand. This clinical trial was prospectively registered at ClinicalTrial.gov (Clinical trial registration number: NCT03669107). Additionally, ethical approval was obtained from the Institutional Research Committee (IRB) of Rajavithi Hospital with the registration number: 61073. 

After approval from the IRB was obtained, eligible patients were invited to participate on the day of admission by one of the research team members. The study information was explained in detail and patients who provided written informed consent were enrolled. Subsequently, web-based computer-generated randomization (www.Randomization.com) using the block-of-four method was performed by an independent investigator. Patients were assigned randomly in a one-to-one ratio to either receive gum-chewing (intervention group) or standard postoperative care (control group). Randomization numbers were stored in sequentially numbered sealed-opaque envelopes. After the surgery, the assigned intervention was revealed and implemented to the patients by the responsible nursing staff in the gynecologic oncology service ward. The nature of the study did not permit complete blinding to patients and nursing staff after the assignment of the intervention. However, patients and nursing staff were educated to keep the group assignment in secret. Therefore, the clinicians, outcome assessors, and investigators were blinded to the treatment assignment throughout the conduct of the study.

*Participants*

Female patients diagnosed with gynecological cancers, such as cervical, uterine, ovarian, fallopian tube, or peritoneal cancer, and scheduled for comprehensive surgical staging surgery were recruited and invited to join in this study. Inclusion criteria were female patients aged 18 to 60 years, good consciousness, suspected or had histologically confirmed gynecological cancers including cervical, uterine, or ovarian/fallopian tube/peritoneal cancer, and were scheduled to undergo surgery. Patients were excluded if they had synchronous cancers, required emergency surgery, unable to chew, got braces or dentures, mint allergy, known history of gastrointestinal disease, thyroid disease, chronic constipation (defined as fewer than 3 defecations per week for at least 3 months)(Gray, 2011), or poor cognitive function, risk of choking or dysphagia due to a pre-existing neurological disorder (such as after a stroke), prior bowel surgery, prior abdominal irradiation, prior neoadjuvant chemotherapy, required bowel anastomosis or abdominal visceral organs surgical approaches regarding to the debulking surgery, developed severe postoperative complication (e.g. massive intraoperative blood loss, intraluminal bowel injury, and severe infection), need for post-operatively intensive care for more than 24 hours, and need for nasogastric tube drainage beyond the first postoperative morning.

*Intervention*

Patients in the intervention group of this study received gum-chewing after surgery with the instruction to chew gum for 30 minutes starting on the first postoperative morning then every 8 hours and continue as instructed until the first passage of flatus. Patients in the control group of the study were instructed not to use gum-chewing but still received standard postoperative care. The gum-chewing used was Clorets^®^ Mint Tab Sugar-free with original mint flavor tablets (Mondelez International, Thailand Co., Ltd). Ingredients include natural and artificial flavors (sorbitol 97%, aspartame 0.4%, sucralose 0.15%), gum base, emulsifier (INS473) and anticaking agent (INS511). 

*Study procedures*

All eligible patients who were invited and willing to participate in this study provided written informed consent. At this time, baseline characteristics and clinical data including height, weight, body mass index (BMI), underlying disease, and tumor characteristics were recorded by investigators. The same standard protocol of pre-, peri-, and postoperative management was used for all patients. On the day before surgery, patients received a clear liquid diet and bowel preparation with soap-suds enema (SSE), sodium phosphate solution (Swiff^®^), or polyethylene glycol (PEG)-based solution until midnight. Prophylactic intravenous antibiotics (ceftriaxone 2 gm or clindamycin 900 mg if penicillin-allergy) were administrated intra-operatively at the induction of anesthesia. Consultant anesthesiologists who used the same anesthetic technique provided general anesthesia with or without epidural anesthesia. Then, surgery was performed by the gynecologic oncologist staff of the Department of Obstetrics and Gynecology, Rajavithi Hospital. The type of surgical incision and procedures were based on the patient disease. However, all patients underwent their surgical staging procedure by the same standard manner of gynecological cancer care.

The postoperative protocol included standard pain medication and the prokinetic agent as an anti-emetic such as metoclopramide, and stress-induced gastritis prophylaxis in the form of H2 blockers. Intravenous fluid 3,000 ml was given during the first 24 hours. The nasogastric tube was removed immediately after surgery and following the removal of a urinary catheter on the first postoperative morning. Regular oral paracetamol was provided, and additional opioid or nonsteroidal analgesia, as well as other anti-emetic agents, were prescribed if they required. Any additional analgesic and anti-emetic agents were recorded. Also, early mobilization was encouraged after assuming a sitting position in bed for 10 minutes to prevent hypotension starting from 24 hours after surgery.

The postoperative feeding regimen was standardized for all study patients. Beginning with 30-60 ml of water was started on the first postoperative day totaling to at least 1 liter per day until the first passage of flatus. After passing flatus, a clear liquid diet and soft diet were allowed and advanced to a regular diet as tolerated or the passage of feces. To ensure compliance, the administration of intervention was implemented by nursing staff and recorded in the case report from. Patients allocated to the gum-chewing group began chewed gum on the first operative morning under the supervision of nursing staff. Each chewing session lasted 30 minutes and then continue every 8 hours while awake. Patients were also instructed to not chew gum during the night or bedtime. All gum-chewing patients completed their course of gum-chewing until the first passage of flatus. For the control group, patients received noting by mouth, except for the postoperative feeding regimen as previous mentioned, until the return of bowel function which defined as the passage of flatus in the absence of vomiting or abdominal distention. An outcome assessor, who was blinded from the treatment assignment, assessed every patient’s bowel sound using a stethoscope three times per day beginning at 24 hours after surgery. All patients were instructed to notify the nursing ward staff immediately when the first passage of flatus, a bowel movement, and defecation has occurred. 

Patients in either group who were unable to tolerate their diet were given noting by mouth and received intravenous hydration until the resolution of their symptoms. A nasogastric tube was placed for intractable nausea, vomiting, or symptomatic abdominal distention. Other postoperative complications, such as hemorrhage, infection, and deep vein thrombosis, were also monitored throughout the hospitalization. Standard criteria for discharge, including stable vital signs with no fever for at least 24 hours, the ability to ambulate without assistance, the ability to tolerate regular diet without vomiting, normal urination and defecation, and no complications after surgery, were used for all study patients. 

*Outcome measurement*

The primary outcome of this study was the time to first passage of flatus. The time at the end of the operation was defined as the zero (0) hour. The secondary outcome included the time to first hearing of normal bowel sounds or movement, time to first defecation, time to first walk, postoperative nausea and vomiting, anti-emetic drugs requirement, any potential side-effects of gum-chewing (e.g., dry mouth, choking, aspiration, and jaw pain), and the length of hospital stay.

The time to first bowel sound or movement was defined as the time to the first hear bowel sound during routine postoperative treatment. The time to first walk was measured form the end of surgery (when patients woke up from anesthesia) until the patients able to ambulate without assistance. Ileus symptoms were categorized as “mild” if they resolved spontaneously within a few days with only observation and basic support, “moderate” if vomiting persisted and reinsertion of the nasogastric tube was required, and “severe” if symptoms persisted for greater than two days or resisted treatment. 

The time to first bowel sound was evaluated three times daily by the outcome assessors who were blinded to the treatment assignment. This measure was starting at 24 hours after surgery until the first bowel sound was noticed. 

Collected data included patient characteristics, tumor characteristics, surgical procedure, surgical complications, stage of the disease, and postoperative outcomes including time to first flatus, time to first bowel sound, time to first defecation, time to first walk, length of hospital stay, additional analgesic and anti-emetic drugs, and ileus symptoms. Patient privacy was protected by coding and processing all data anonymously. 

*Statistical analysis*

The sample size calculation was based on the primary outcome, time to passage of the first flatus, from the study of Ertas et al. that performed in patients who underwent complete surgical staging for gynecological cancers by using the formula for the test of difference in two independence means (Ertas et al., 2013). The mean time interval to the passage of the first flatus and standard deviation were 43.6 ± 14 and 34 ± 11.5 hours in the control group and the gum-chewing group, respectively. With applying 0.05 alpha level and 90% power, the sample size was calculated to be 28 patients for each treatment group. After adjustment for a drop-out or withdrawal rate of 30%, a minimum of 40 patients in each treatment group was required. 

Statistical analysis was performed using the Stata software package, version 15.1 (Stata Corp, College Station, Texas, USA). All analyses of the effect of gum-chewing were based on the principle of intention-to-treat-basis, and all eligible patients were included in the group according to which they were randomized, independently of whether they received the assigned treatment. 

Descriptive statistics for continuous variables with normal distribution were analyzed by using the Student’s t-test and expressed in the mean ± standard deviation. Whereas the descriptive statistics for continuous variables with non-normal distribution were analyzed by using the Mann-Whitney U test and expressed in median and range. Categorical variables were assessed using the Pearson’s chi-square test or Fisher’s exact test and expressed as the number of cases and in the percentage (%) form. Time to first flatus, time to first bowel sound, time to first defecation, and time to first walk were also analysis with the use of Kaplan-Meier methods and compared among the two treatment groups using the log-rank test. A two-sided p-value of less than 0.05 will be considered to indicate statistical significance for all tests. 

## Results

**Figure 1.**
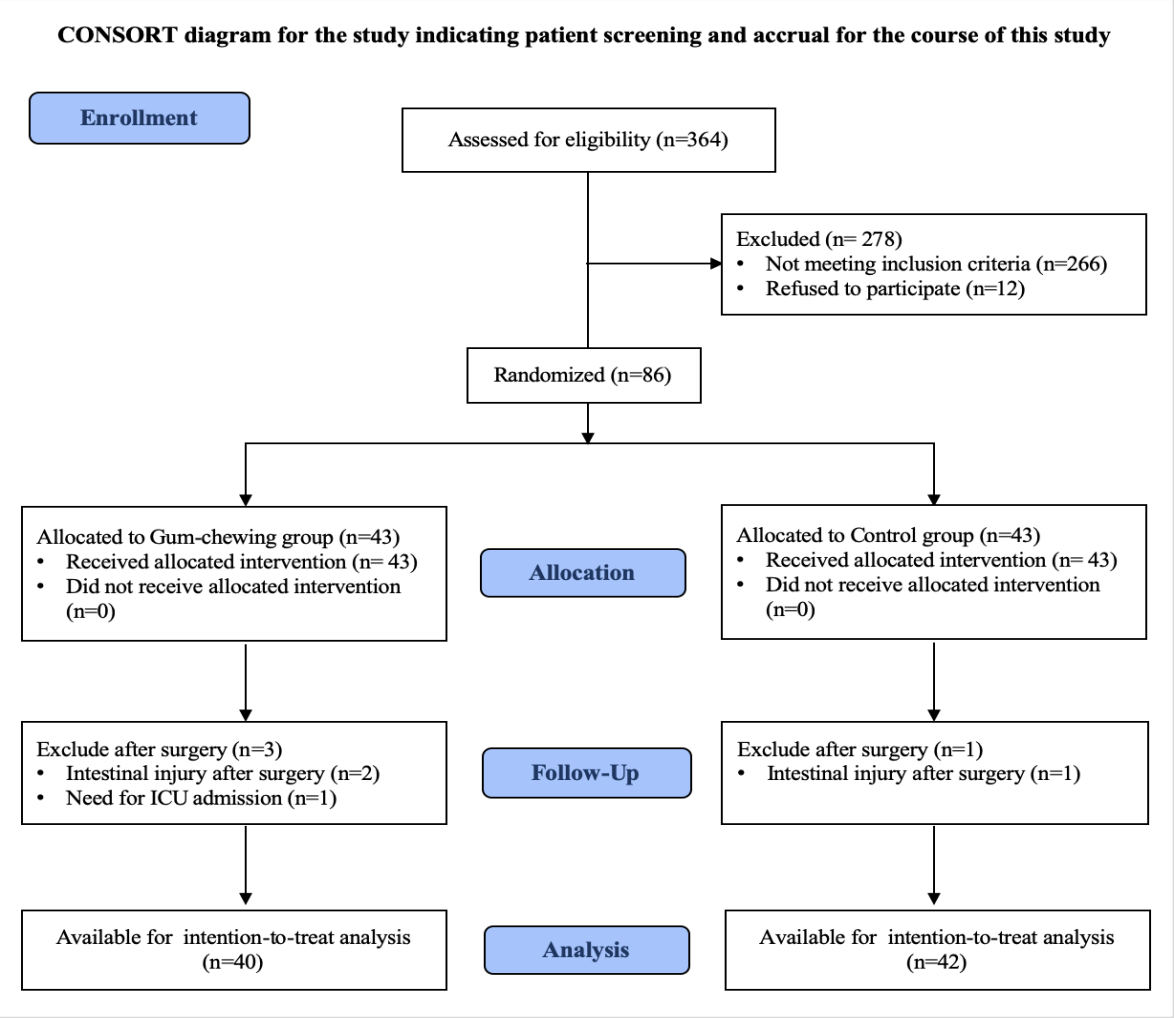
Participant Flow Diagram after Randomization to either the Gum-chewing Group or the Control Group

From 1^st^ October 2018 to 30^th^ June 2019, a total of 364 gynecological cancer patients who had been scheduled for elective surgery at Rajavithi Hospital were enrolled and assessed for eligibility. Of those patients assessed, 278 were excluded primarily because of existing exclusion criteria (n=266) and refused to participate (n=12). Therefore, 86 patients were included in this randomized controlled trial and were equally assigned randomly to the gum-chewing group and the control group of 43 patients in each group. Four patients were excluded after randomization because they no longer fulfilled the inclusion criteria (3 in the gum-chewing group and 1 in the control group). Finally, 40 patients in the gum-chewing group and 42 patients in the control group were included in the intention-to-treat analysis. The participant flow diagram and the reasons for exclusion before and after randomization are shown in Figure 1.

Baseline demographic data and clinical characteristics of the included patients in the two groups were similar and presented in Table 1. The mean age of patients was 48.2 ± 8.3 years and more than half (56.1%) of the patients were postmenopausal women. In both groups, endometrial cancer was the most common indication for comprehensive staging surgery (37.5 % in the gum-chewing group and 38.1% in the control group). The most common histopathology is adenocarcinoma (79.3%) and most patients had an early stage of cancer (63.5%). 

Surgical characteristics compared between the gum-chewing group and the control group are summarized in Table 2. Patients in both groups had similar surgical characteristics including type of surgery, type of surgical procedure, mean duration of operation, anesthesia technique, estimated blood loss, rate of lymphadenectomy, omentectomy, appendectomy, abdominal adhesiolysis, and required for blood transfusion. Almost all patients (90.2%) underwent laparotomy and the most common type of surgical procedure was hysterectomy with bilateral salpingo-oophorectomy (73.2%). Nearly half of all patients (36.6%) had completed lymphadenectomy included pelvic and para-aortic lymph nodes. Interestingly, almost all patients (92.7%) received general endotracheal anesthesia with an epidural block.

Gum-chewing was well tolerated, and all patients completed their course of gum-chewing until the passage of the first flatus. Moreover, no adverse events such as dry mouth, choking, aspiration, and jaw pain were observed related to gum-chewing. The postoperative clinical outcomes of the study are shown in Table 3. In either group, there were no complications such as fever (defined as temperature > 38ºC), re-operation, deep vein thrombosis, and readmission after hospital discharge. 

**Figure 2:**
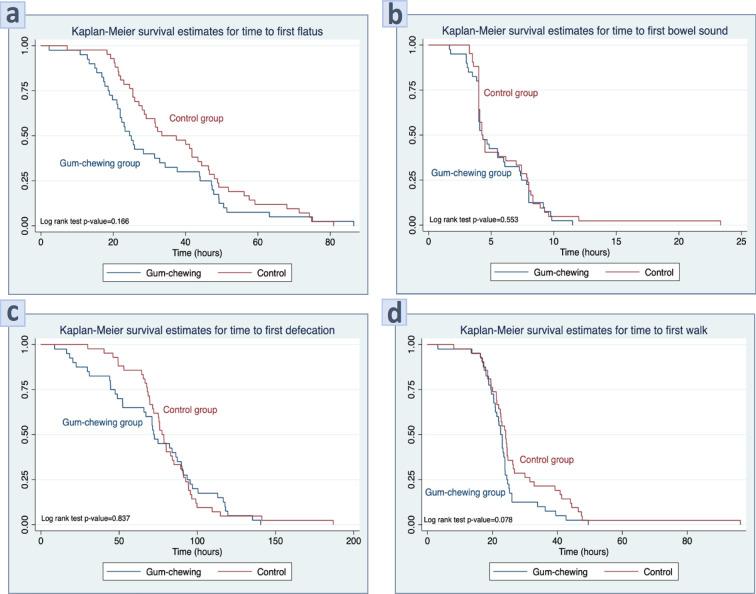
Kaplan-Meier Represents of (a) Time to Passage of First Flatus, (b) Time to First Postoperative Bowel Movement, (c) Time to First Defecation, and (d) Time to First Walk

We found a significantly shorter interval between the end of surgery and the passage of the first flatus in the gum-chewing group compared with the control group (24.7 hours compared with 35.4 hours, median difference 12.4 (95%CI; 0.98,23.85) hours, p=0.025). In addition, the length of hospital stay was significantly shorter in the gum-chewing group (3.0 days compared with 3.5 days, median difference 0.87 (95%CI; 0.14,1.60) days, p=0.023). It seems to be that the time to first bowel sound, time to first defecation, and time to first walk were shorter in the gum-chewing group than in the control group. However, these differences were not statically significant (p>0.05). These findings are also shown graphically in the Kaplan-Meier curves in Figure 2. 

The incidence of postoperative nausea, vomiting, abdominal distention, and ileus symptoms were comparative in both two groups. Mild ileus symptoms were observed in 14 patients (35%) in the gum-chewing group compared to 18 patients (42.8%) in the control group. These patients were treated by fasting, intravenous fluid administration to correct any underlying electrolyte abnormality, and anti-emetic pills. It was detected that there were no moderate ileus symptoms in the gum-chewing group, but there were seen in 2 patients (4.8%) in the control group because they required the insertion of nasogastric tubes for gastric decompression. However, there were no patients who progressed to severe ileus symptoms or bowel obstruction. Also, all patients were able to tolerate oral intake before hospital discharge.

Furthermore, we also tested the influence of postoperative gum-chewing on the additional need for analgesic and anti-emetic drugs on the day of surgery. The result showed that patients who were assigned in the gum-chewing group were required additional analgesic drugs and anti-emetic drugs at a similar rate when compared with the control group (82.5% compared with 76.2%, p=0.481 and 5.0% compared with 7.1%, p=1.000).

## Discussion

Postoperative bowel ileus (POBI) remains a source of morbidity and a major determinant of the length of hospital stay after gynecological cancer surgery. It is a psychological problem and an economic burden for gynecological cancer patients, as well as a health-care system. For these reasons, strategies to reduce these post-operative sequelae and hospitalization length are of great importance. Many mandatories such as motility agents, early postoperative feeding, physical therapy, including an implement postoperative protocol as ERAS programs have been investigated in clinical trials, but are not routinely used because of their limited clinical efficacy and not strongly recommendations (Charoenkwan et al., 2007; Purkayastha et al., 2008; Traut et al., 2008; Johnson and Walsh, 2009; Gungorduk et al., 2017; Ljungqvist et al., 2017; Nelson et al., 2019).

From a mechanistic and physiologic viewpoint, it is plausible that gum-chewing could have beneficial effects on bowel recovery. The proposed mechanism is through a sham feeding pathway in which activation of the cephalic-vagal phase of digestion and leads to upregulation of gastric hormones and motility (Konturek, 2003; Arosio et al., 2004). Thus, we conducted this randomized clinical trial for assessing the effect of gum-chewing on gastrointestinal function recovery in terms of time to first flatus, time to first bowel sound or bowel movement, time to first defecation, time to first walk, and length of hospital stay in patients undergoing comprehensive surgery for gynecological cancers.

POBI occurs due to the drop in intestinal movement and the reduction of the activity of parasympathetic nervous systems. It occurs in cases of opioid administration and abdominal operations, especially in extensive surgeries with extremely manipulation and transiently contributes to deferring bowel peristalsis. In the present study, we selected the inclusion criteria as the patients undergoing comprehensive surgery for gynecological cancers because from the previous literature have shown that the incidence of POBI in patients treating with comprehensive surgical staging for gynecological cancers was high (10.6 to 50.0%) and it might be sufficient for showing a significant difference result (Fujita et al., 2005; Tabata et al., 2010). The increasing incidence of ileus is due to extensive surgery including systematic pelvic and para-aortic lymphadenectomy which exist in surrounding areas of major vessels. If all surrounding areas of major vessels were to be dissected, many nerve ganglions would be resected simultaneously, and consequently, the paralytic ileus would occur.

As a matter of fact, the practices which are accessible, inexpensive, and consistent after abdominal surgeries should also utilize the postoperative concerning in patients in order to reduce the recovery period and length of time to discharge from the hospital. Gum-chewing is one kind of manner as an artificial or sham feeding that hasten bowel movements in a short time in various studies (Purkayastha et al., 2008; Abd-El-Maeboud et al., 2009; Ertas et al., 2013; Craciunas et al., 2014; Jernigan et al., 2014; Xu et al., 2018). Consequently, we have taken up the gum-chewing as an intervention in this study. Besides, we have chosen a sugar-free gum in this study because the sugar-free gum substitutes, such as sorbitol and xylitol, can improve gastrointestinal function by causing a non-stimulant laxative effect which may influence bowel motility in these patients (Tandeter, 2009).

Ileus resolution is habitually defined by the passage of flatus (gas) or feces or both. These are signs that intestinal function is being restored to normal, and the endpoints of POBI are usually measured (Vather et al., 2013). Therefore, we determined the definition of gastrointestinal function recovery in this study by the first passage of flatus, bowel movement postoperatively, and the first defecation. Because the measurement of these signs is subjective, especially the auscultation of the bowel movement. To increase the validity of the outcome measurements, we have trained the outcome assessors to measure this outcome in the same way and integrated other components for assessing the postoperative parameter of gastrointestinal function such as abdominal distention, nausea, and vomiting after surgery. Additionally, because the postoperative ileus is a major determinant of length of hospital stay, this endpoint was also captured as part of the secondary analysis in the present study.

The results of the present study have indicated that gum-chewing after gynecological cancer surgery can significantly promote the gastrointestinal function recovery by accelerating the time to the passage of the first flatus and shorter the length of hospital stay. These findings are clinically relevant and important for gynecologic oncologist surgeons because they place the patients at a small risk for POBI with the generally fast recover of normal bowel motility and passage of the first flatus owing to the beneficial effect of post-operative gum-chewing. Also, a shortening in the length of hospital stays related to gum-chewing towards a reduced in the patient’s morbidity and health care costs. 

Ertas et al., (2013) performed a randomized controlled trial to investigate the effect of post-operative gum-chewing on the bowel motility after surgery for malignant gynecological conditions. The result reported a significantly shorter interval of time to the passage of the first flatus (34.0 ± 11.5 vs 43.6 ± 14.0 hours, p<0.001) and the length of hospital stay (5.9 ± 1.0 vs 7.0 ± 1.4 days, p<0.001) in the gum-chewing group compared to the control group. Similarly, the present study demonstrated the same results. The median time to the passage of first flatus in the gum-chewing group and the control group was 24.7 and 35.4 hours, respectively. Whereas the median length of hospital stay in the gum-chewing group and the control group was 3.0 and 3.5 days, respectively. However, it interesting to note that the median time to first flatus and the median length of hospital stay of our study were shorter than Ertas’s study. The possible explanation may be that most of all patients (92.7%) in our study receive epidural anesthesia techniques. Cochran review demonstrated that the type of anesthesia might have an impact on the recovery of gastrointestinal function, especially the use of epidural local anesthesia. Such, it could be reduced postoperative gastrointestinal paralysis in terms of time to passage of flatus and first bowel movement when compared with a systemic opioid in general anesthesia (Guay et al., 2016). 

Nowadays, no adverse effects have been reported after the use of gum-chewing to stimulate sham feeding in patients after surgery (Abd-El-Maeboud et al., 2009; Ertas et al., 2013; Craciunas et al., 2014; Jernigan et al., 2014; Xu et al., 2018; Campbell et al., 2019). Similarly in the present study, all patients in the gum-chewing group were well tolerated and completed their course of gum-chewing until the passage of the first flatus. Also, no adverse events such as dry mouth, choking, aspiration, and jaw pain, were observed related to the gum-chewing. Summing it up, the present study’s results take these findings to the next logical step, demonstrating that gum is a safe, pleasant, and effective intervention to prevent POBI. 

For clinical application, chewing gum should be added to the postoperative care guideline. Because it was associated with shorter gastrointestinal function recovery and would lead to a reduction in length of hospital stay for approximately one day. The benefit of earlier discharge from the hospital will lead to cost-saving for 1,000 Thai Baht (THB) per one person. Additionally, gum-chewing has a low cost when compared with pharmacologic management to prevent ileus, such as u-opioid receptor antagonists, ghrelin receptor agonists, and serotonin receptor agonists. The US Food and Drug administration approved alvimopan, a novel oral peripherally acting u-opioid receptor antagonist, for use after bowel surgery (Herzog et al., 2006; Erowele, 2008). But the perioperative course of alvimopan cost is approximately 3,000 THB whereas a pack of 8 tablets of the sugar-free gum-chewing used in the present study sells for 15 THB. Therefore, another interesting issue that would recommend for studying in future research is the cost-effective analysis of gum-chewing in prevention of POBI after abdominal surgery.

The present study had several strengths which included the fact that it was a prospective randomized clinical trial that decreased the selection bias and that the two groups had similar demographic and surgical characteristics reflecting all patients have the equivalent prognosis and a good randomization process. Moreover, the study was performed at a single institution by the same manner of surgical procedure which probably increases the validity of our results due to minimizing the variation of operative techniques and treatment protocols.

On the other hand, the present study had several limitations. First, our study had a small sample size involving a limited number of patients which might be a reason for not showing a significant difference in other outcomes, such as time to first bowel sound, time to first defecation, and time to first walk. However, it was adequately powered to answer the question of whether gum chewing has a substantial impact on postoperative gastrointestinal function recovery. Second, the study population represented a highly selected group of patients undergoing comprehensive surgery for gynecological cancers without any complications. Patients with more aggressive surgery such as bowel resection or re-anastomosis were excluded, and these patients were more likely to experience a POBI after surgery. Also, a limited sampling population from one hospital and highly specific inclusion criteria will lead to no external validation with other populations. Third, we did not have a placebo group, thus, we do not know whether there were any placebo effects that may occur during the outcome measure. Fourth, the patients were not blinded, this may have affected the effect of gum-chewing to a small extent. Finally, the best type of gum and the optimal amount remain unclear. Thus, further trials with larger sample size, blinded, placebo-controlled, and multicenter, are required to address this issue before adding the gum-chewing to standard clinical practice guidelines for postoperative care after gynecological cancer patients. 

In conclusion, Gum-chewing was associated with early recovery of gastrointestinal function in patients undergoing comprehensive surgery for gynecological cancer. The benefits of this approach included faster time to flatus and shorter hospital stay resulting in decreased patient’s morbidity and health care cost. Also, it is an inexpensive and physiologic intervention that appears to be reasonably safe and should be recommended as an adjunct in postoperative care of gynecological cancer surgery.

**Table 1:** Baseline Demographic and Clinical Characteristics Compared between the Gum-chewing and the Control Group

Characteristic	Gum group (n=40)	Control group (n=42)	Total (n=82)	P value
Age (years), mean ± SD	48.9 ± 7.3	47.5 ± 9.1	48.2 ± 8.3	0.431
BMI group, n (%)				0.245
Underweight	5.0 (12.5)	1.0 (2.4)	6.0 (7.3)	
Normal	17.0 (42.5)	19.0 (45.2)	36.0 (43.9)	
Overweight	13.0 (32.5)	12.0 (28.6)	25.0 (30.5)	
Obesity	5.0 (12.5)	10.0 (23.8)	15.0 (18.3)	
Underlying disease, n (%)				
Diabetes mellitus	6.0 (16.3)	14.0 (33.3)	20.0 (24.4)	0.053
Hypertension	11.0 (27.5)	10.0 (23.8)	21.0 (25.6)	0.702
Dyslipidemia	5.0 (12.5)	4.0 (9.5)	9.0 (11.0)	0.735
Kidney disease	0.0 (0.0)	2.0 (4.8)	2.0 (2.4)	0.494
Other	4.0 (10.0)	12.0 (28.6)	16.0 (19.5)	0.05
Menopause, n (%)				0.522
Pre-menopausal	19.0 (47.5)	17.0 (40.5)	36.0 (43.9)	
Post-menopausal	21.0 (52.5)	25.0 (59.5)	46.0 (56.1)	
Previous abdominal surgery, n (%)	11.0 (27.5)	16.0 (38.1)	27.0 (32.9)	0.307
Type of cancer, n (%)				0.717
Ovarian cancer	14.0 (35.0)	11.0 (26.2)	25.0 (30.5)	
Endometrial cancer	15.0 (37.5)	16.0 (38.1)	31.0 (37.8)	
Cervical cancer	8.0 (20.0)	9.0 (21.4)	17.0 (20.7)	
Other	1.0 (2.5)	4.0 (9.2)	5.0 (6.1)	
Histopathology, n (%)				1
Adenocarcinoma	32.0 (80.0)	33.0 (78.6)	65.0 (79.3)	
SCCA	4.0 (10.0)	4.0 (9.5)	8.0 (9.8)	
Sarcoma	1.0 (2.5)	1.0 (2.4)	2.0 (2.4)	
Other	3.0 (7.5)	4.0 (9.5)	7.0 (8.5)	
FIGO stage of cancer, n (%)				0.98
Early stage (I-II)	19 (63.3)	21 (63.6)	40.0 (63.5)	
Advanced stage (III-IV)	11 (36.7)	12 (36.4)	23.0 (36.5)	

Abbreviations, BMI, Body Mass Index; kg/m^2^, kilograms per meter square; SCCA, squamous cell carcinoma; FIGO, International Federation of Gynecology and Obstetrics; SD, standard deviation

**Table 2:** Surgical Characteristics Compared between the Gum-chewing and the Control Group

Characteristic	Gum group (n=40)	Control group (n=42)	Total (n=82)	P value
Type of surgery, n (%)				1.000
Laparotomy	36.0 (90.0)	38.0 (90.5)	74.0 (90.2)	
Laparoscopy	4.0 (10.0)	4.0 (9.5)	8.0 (9.8)	
Surgical procedure, n (%)				0.851
Salpingo-oophorectomy	4.0 (10.0)	3.0 (7.1)	7.0 (8.5)	
Hysterectomy with salpingo-oophorectomy	28.0 (70.0)	32.0 (76.2)	60.0 (73.2)	
Radical hysterectomy	6.0 (15.0)	5.0 (11.9)	11.0 (13.4)	
Modified hysterectomy	2.0 (5.0)	1.0 (2.4)	3.0 (3.7)	
Other	0.0 (0.0)	1.0 (2.4)	1.0 (1.2)	
Lymphadenectomy, n (%)				0.776
Pelvic lymph node	1.0 (2.5)	1.0 (2.4)	2.0 (2.4)	
Para-aortic lymph node	5.0 (12.5)	6.0 (14.3)	11.0 (13.4)	
Both	17.0 (42.5)	13.0 (30.9)	30.0 (36.6)	
Omentectomy, n (%)	24.0 (60.0)	22.0 (52.4)	46.0 (56.1)	0.487
Appendectomy, n (%)	3.0 (7.5)	3.0 (7.1)	6.0 (7.3)	1
Abdominal adhesiolysis, n (%)	18.0 (45.0)	12.0 (28.6)	30.0 (36.6)	0.123
Duration of operation(hours), mean ± SD	2.4 ± 0.8	2.4 ± 0.8	2.4 ± 0.8	0.79
Anesthesia technique, n (%)				0.676
With epidural block	38.0 (95.0)	38.0 (90.5)	76.0 (92.7)	
Without epidural block	2.0 (5.0)	4.0 (9.5)	6.0 (7.3)	
Blood loss (ml), median (range)	325	325	325	0.852
	(30.0, 4,000.0)	(20.0, 2,700.0)	(20.0, 4,000)	
Required blood transfusion, n (%)	3.0 (7.5)	2.0 (4.8)	5.0 (6.1)	0.672

Abbreviations, ml, milliliters; SD, standard deviation

**Table 3:** Postoperative Clinical Outcomes Compared between the Gum-chewing and the Control Group

Variables	Gum group (n=40)	Control group (n=42)	P value
Primary outcome			
Time to first flatus time (h), median (range)	24.7 (2.2, 86.5)	35.4 (7.2, 80.9)	0.025*
Secondary outcomes			
Time to first bowel movement (h), median (range)	4.3 (1.7, 11.5)	4.3 (3.2, 23.3)	0.501
Time to first defecation time (h), mean ± SD	72.6 ± 34.3	79.6 ± 27.4	0.224
Time to first walk (h), median (range)	22.7 (3.2, 49.5)	24.1 (8.1, 96.3)	0.157
Hospital stay (d), median (range)	3.0 (1.0, 8.8)	3.5 (1.8, 50.0)	0.023*
Postoperative nausea, n (%)	6.0 (15.0)	5.0 (11.9)	0.681
Postoperative vomiting, n (%)	0.0 (0.0)	5.0 (11.9)	0.055
Abdominal distention, n (%)	11.0 (27.5)	18.0 (42.9)	0.146
Ileus symptoms, n (%)			0.331
Mild	14.0 (35.0)	18.0 (42.9)	
Moderated	0.0 (0.0)	2.0 (4.8)	
Severe	0.0 (0.0)	0.0 (0.0)	
Additional analgesic drug, n (%)	33.0 (82.5)	32.0 (76.2)	0.481
Additional antiemetic drug, n (%)	2.0 (5.0)	3.0 (7.1)	1

Abbreviations, h, hours; d, days; SD, standard deviation
